# EGFR-Based Targeted Therapy for Colorectal Cancer—Promises and Challenges

**DOI:** 10.3390/vaccines10040499

**Published:** 2022-03-24

**Authors:** Balakarthikeyan Janani, Mayakrishnan Vijayakumar, Kannappan Priya, Jin Hee Kim, D. S. Prabakaran, Mohammad Shahid, Sameer Al-Ghamdi, Mohammed Alsaidan, Nasraddin Othman Bahakim, Mohammad Hassan Abdelzaher, Thiyagarajan Ramesh

**Affiliations:** 1 Department of Biochemistry, PSG College of Arts and Science (Autonomous), Bharathiar University, Coimbatore 641014, Tamil Nadu, India; jananijana025@gmail.com; 2 Department of Integrative Bioscience and Biotechnology, College of Life Sciences, Sejong University, 209 Neugdong-ro, Gwangjin-gu, Seoul 05006, Korea; marulbiochem@rediffmail.com (M.V.); jhkim777@sejong.ac.kr (J.H.K.); 3 Department of Radiation Oncology, College of Medicine, Chungbuk National University, Chungdae-ro 1, Seowon-gu, Cheongju 28644, Korea; prababio@chungbuk.ac.kr; 4 Department of Biotechnology, Ayya Nadar Janaki Ammal College (Autonomous), Srivilliputhur Main Road, Sivakasi 626124, Tamil Nadu, India; 5 Department of Basic Medical Sciences, College of Medicine, Prince Sattam Bin Abdulaziz University, Al-Kharj 11942, Saudi Arabia; dr.shahid90@yahoo.com (M.S.); nasroden2010@gmail.com (N.O.B.); abdelzaher_gerga@yahoo.com (M.H.A.); 6 Family and Community Medicine Department, College of Medicine, Prince Sattam Bin Abdulaziz University, Al-Kharj 11942, Saudi Arabia; sh.alghamdi@psau.edu.sa; 7 Internal Medicine Department, College of Medicine, Prince Sattam Bin Abdulaziz University, Al-Kharj 11942, Saudi Arabia; dr.saidan@hotmail.com; 8 Department of Medical Biochemistry, Faculty of Medicine, Al-Azhar University (Assiut Branch), Assiut 71515, Egypt

**Keywords:** colorectal cancer, EGFR, nanocarriers, nanomedicine, cetuximab

## Abstract

Colorectal carcinoma (CRC) is the most lethal and common form of cancer in the world. It was responsible for almost 881,000 cancer deaths in 2018. Approximately 25% of cases are diagnosed at advanced stages with metastasis—this poses challenges for effective surgical control and future tumor-related mortality. There are numerous diagnostic methods that can be used to reduce the risk of colorectal carcinoma. Among these, targeted nanotherapy aims to eliminate the tumor and any metastasis. Active targeting can increase the effectiveness and quantity of drugs delivered to the target site. Antibodies that target overexpressed receptors on cell surfaces and indicators are coupled with drug-loaded carriers. The major target receptors of chemotherapeutic drugs delivery include VEGFR, EGFR, FGFR, HER2, and TGF. On account of its major and diverse roles in cancer, it is important to target EGFR in particular for better tumor selection, as EGFR is overexpressed in 25 to 82% of colorectal carcinoma cases. The EGFR monoclonal immunoglobulins cetuximab/panitumumab can thus be used to treat colorectal cancer. This review examines carriers that contain cetuximab-conjugated therapeutic drugs as well as their efficacy in anticancer activities.

## 1. Introduction

Colorectal cancer (CRC) is the most lethal and common form of cancer in the world. It was responsible for almost 881,000 deaths from cancer [1]. The primary causes of CRC are not known but may involve lifestyle, viruses, smoking, and environmental hazards. Mutation of the adenomatous polyposis coli (APC) gene is likely to occur during the initial stage of CRC development [2]. The advancement of screening technologies, such as the fecal occult blood test, colonoscopy, and colonography, enable the early detection of colorectal cancer. The emergence of less-invasive surgical methods such as endoscopic, laparoscopic, and robotic procedures has contributed to a reduction in the total number of patients requiring operation for resectable colorectal cancer [3]. There are several diagnostic methods available to reduce the incidence of CRC. However, approximately 25% of CRCs are detected at an advanced stage with metastasis. Furthermore, 20% of cases may go on to develop metachronous metastasis. This poses challenges in surgical control and subsequent cancer-related mortality [1]. Controlling the disease is still challenging in patients with advanced-stage CRC, and they require intensive treatments such as chemotherapy with irinotecan or oxaliplatin, signal inhibitors, and antibodies to achieve a satisfactory outcome [3]. Since the primary goal of CRC treatment is to completely eradicate the tumor and metastasis, which is most often accomplished by invasive surgery on account of varying tumor responses to different treatment techniques, it is crucial to choose the optimal treatment strategy for CRC. The treatment is chosen for the patient depending on several criteria, including the type of tumor, stage of the disease, patient age, overall patient health, and patient attitude towards life [2,4]. Despite further current knowledge of the molecular and cellular aspects of cancer, existing treatments still focus on systemic chemo- and radiotherapy. Broad distribution is a common problem with these regimens, which commonly results in inadequate dosage for the treatment of the tumor and/or the production of harmful side effects in normal tissue [4].

**Figure 1:**
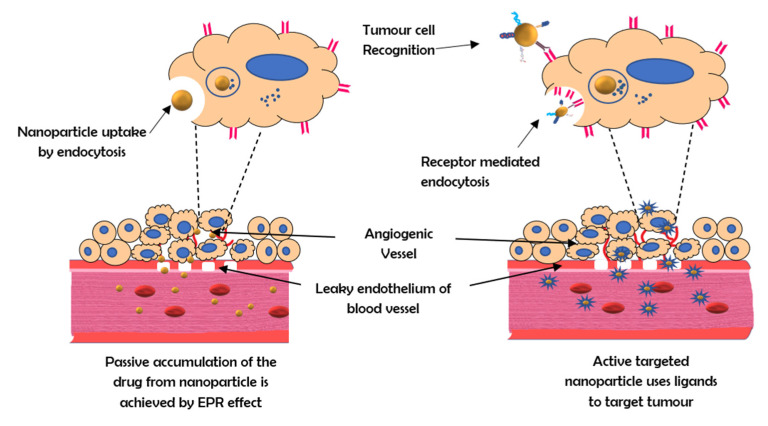
The mechanism of active and passive targeting by nanocarriers.

It is possible to target specific changes in cancer cell biology that are highly upregulated, when compared to those of the healthy surrounding cells and tissues, by introducing a targeting moiety (ligand, antibody, or peptide) into the nanoparticle system [5]. The addition of a targeting moiety also enhances drug absorption through receptor-mediated endocytosis, which is an active mechanism requiring a much lower concentration gradient across the plasma membrane than basic endocytosis (Figure 1) [6]. With the help of active targeting, both the quantity of drug delivered and therapeutic efficiency can be enhanced while decreasing the side effects of the drug [7].

## 2. Receptors Used for Targeted Therapy

The strategy of major nanoparticular anti-tumor targeting research is to use antibodies to target disease-associated surface markers on cells. These markers, often receptors, are typically elevated or expressed in particular tumor-associated cells. These receptors can be targeted to deliver chemotherapeutic drugs. They include EGFR, VEGFR, FGFR, HER2, and TGF-b.

1. EGFR: The epidermal growth factor receptor (EGFR) is a receptor tyrosine kinase belonging to the ErbB family of proteins. Ligand binding is required to activate the tyrosine kinase domain. This activates signaling pathways responsible for cell proliferation, angiogenesis migration, continued existence, and adhesion. Since these pathways are essential for the survival of cancer cells, EGFR is a valuable target in the treatment of colorectal carcinoma metastases [8].
2. VEGFR: The vascular endothelial growth factor receptor (VEGFR) is a tyrosine kinase receptor. Binding of the ligand vascular endothelial growth factor (VEGF) to this receptor leads to the activation of the receptor and promotes vasculogenesis and angiogenesis [9]. VEGF overexpression is observed in 40–60% of colorectal cancers and is related to cancer recurrence and decreased survival [10].
3. FGFR: In several essential physiological mechanisms such as homeostasis of tissue metabolism, embryonic development, endocrine function, and wound repair, angiogenesis fibroblast growth factor (FGFR) signaling pathways are crucially significant [11]. Therapy against FGFR2 and its specific isoforms are being considered as novel treatment options for colorectal cancer patients. By administering shRNA to bind FGFR2, CRC development, invasion, and migration can be reduced [12].
4. HER 2: The type I transmembrane glycoprotein human epidermal growth factor receptor 2 (HER2) is involved in signaling pathways that control cell proliferation, survival, and apoptosis in breast cancer. In 20–25% of breast cancer patients, the HER2 gene is amplified, which is connected to an aggressive phenotype and worse prognosis [13]. The efficacy of HER2-targeted therapy is comparable to that of developing therapeutic options for metastatic colorectal cancer, such as immunotherapy with checkpoint inhibitors and BRAF-directed therapy [14].
5. TGF-β: The signaling pathway that is activated by transforming growth factor-beta (TGF-β) is crucial in the regulation of tissue development, proliferation, differentiation, apoptosis, and homeostasis [15]. TGF-β is also a powerful regulator of cell adhesion, motility, and the composition of the extracellular matrix, all of which are implicated in tumor invasion and metastasis. However, TGF-β signaling also stimulates angiogenesis and immunosuppression. TGF-β signaling breakdown in colorectal cancer cells promotes tumor growth in the early stages, whereas the stimulation thereof may enhance cancer invasion and metastasis. Thus, while TGF-β may be used as a target in nanotherapeutic methods, its dual roles in enhancing and suppressing tumorigenesis require it to be treated in a careful and highly selective manner [16].

## 3. Significance of EGFR as a Target

**Figure 2:**
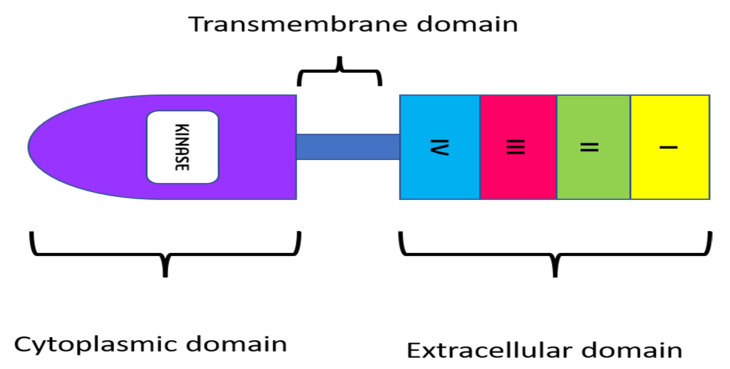
Structure of EGFR receptor.

To design therapeutic approaches, researchers are examining the impact of the epidermal growth factor receptor (EGFR) on the development and prognosis of epithelial malignancies [17,18]. EGFR has emerged as a critical target molecule for enhanced tumor specificity because of its diverse functional roles in cancer [19]. EGFR is a transmembrane tyrosine kinase receptor with a molecular weight of 170 kDa that belongs to the ErbB family of cell membrane receptors. Other receptors in this family, in addition to EGFR (also known as HER1 and ErbB-1), include HER2/c-neu (ErbB-2), HER3 (ErbB-3), and HER4. (ErbB-4). All of these receptors have a cytoplasmic tyrosine kinase-containing domain, a single membrane-spanning region, and an extracellular ligand-binding region (Figure 2) [20]. In several malignancies, such as head and neck carcinomas, EGFR expression, which is normally assessed by immunohistochemistry, has been linked to tumor development and poor survival [21]. However, the importance of EGFR protein expression in other cancers, such as lung carcinomas, remains controversial, as the level of EGFR overexpression may vary anywhere from 25 to 82% in colorectal cancers [22].

In addition to EGFR expression levels, ligand binding to EGFR is important in functions that modulate various aspects of colorectal cancers. ADAM17 is a critical enzyme in the regulation of the release of EGFR ligands, which include EGF, amphiregulin, and heparin-binding EGF [23]. Ligand binding to the extracellular domain phosphorylates the tyrosine kinase domain, activating signaling pathways involved in cell proliferation, angiogenesis, migration, continued existence, and adhesion. Since cancer cells rely on these pathways, EGFR is a useful target in the therapy of colorectal cancer metastases [8]. EGF, transforming growth factor (TGF-), β-cellulin, epiregulin (EREG), epigen (EPGN), amphiregulin (AREG), and heparin-binding EGF are the most common EGFR ligands [24]. Conventional EGFR activation begins when a ligand binds to the extracellular domain, inducing a conformational change that causes monomer EGFRs to dimerize [25]. This brings cytoplasmic regions closer together, enabling autophosphorylation of tyrosine residues in the regulatory region. Autophosphorylated tyrosine residues function as anchors for various genes, including GRB2, SHC, SRC, and PI3K, which recruit and activate the RAS, AKT, and STAT signaling pathways, thereby initiating essential cellular processes [26]. EGFR is often mutated in patients with solid tumors. Overexpression of the EGFR protein and kinase-activating mutations are the two forms of pathogenic EGFR changes found in cancer. The kinase-activating mutations that result in enhanced EGFR tyrosine kinase activity might occur as a result of, or in addition to, anti-EGFR therapy. While these markers can be useful tools in the diagnosis of colorectal cancer, current methods of diagnosis primarily use stool samples and colonoscopies [27]. Colorectal cancer is currently diagnosed using stool samples and colonoscopies. The cancer is classified as either colonic or rectal, depending on its location. Both types of tumors exhibit obvious similarities; however, due to their differences in location, their molecular properties likewise differ. Treatment depends on the tumor’s response to chemotherapy or anti-EGFR monoclonal antibodies [28].

## 4. Monoclonal Antibodies for EGFR Targeted Therapy

Given EGFR’s functional roles in various cellular activities, numerous strategies have been developed to specifically target and inhibit EGFR-mediated effects. Small-molecule tyrosine kinase inhibitors (TKIs) and monoclonal antibodies (mAbs) are two different therapeutic methods used to target EGFR in diverse human cancers [29]. Clinical trials have demonstrated that monoclonal antibodies and TKIs are effective anti-EGFR therapies [30]. The prevention of receptor dimerization, autophosphorylation of the cytoplasmic domain, and downstream signaling take place due to competitive inhibition of EGFR ligands by monoclonal antibodies, which is particularly developed to be directed towards the extracellular region of EGFR [31]. The EGFR monoclonal antibodies that have been approved for clinical use are cetuximab and panitumumab. Their antitumor effect is mediated by specialized structures with various roles. Cetuximab and panitumumab work similarly: They bind to the extracellular domain of EGFR, inhibiting ligand interaction and causing TK internalization and destruction. As a result, they promote apoptosis by blocking EGFR downstream pathways. Anti-EGFR mAbs, especially those of the IgG1 subclass, may also activate the host immune system (antibody-dependent cellular cytotoxicity and complement-mediated cytotoxicity) to destroy the cancer cell. Regardless of the anti-EGFR medicine employed, numerous clinical studies have indicated that cetuximab and panitumumab produce comparable effects [32]. This is mediated by the mAb variable domain fragment (Fv), which is made up of portions of the light and heavy chains of the antibody. Despite their specificity, the production of EGFR mAbs was not without its hurdles. The mAb variable domain fragment (Fv) is a crucial component. It is made up of portions of the antibody’s light and heavy chains. Immunizing mice against EGFR proteins yielded the first mAbs. However, human anti-mouse antibody reactions such as allergic reactions and lower efficacy resulted from these murine antibodies. The combination of a variable murine region with antigenic activity with a constant human region led to the production of fewer allergenic chimeric antibodies such as cetuximab and the Table 1 compiles the list of monoclonal antibodies used in the colorectal cancer treatment [33].

### Cetuximab

**Figure 3:**
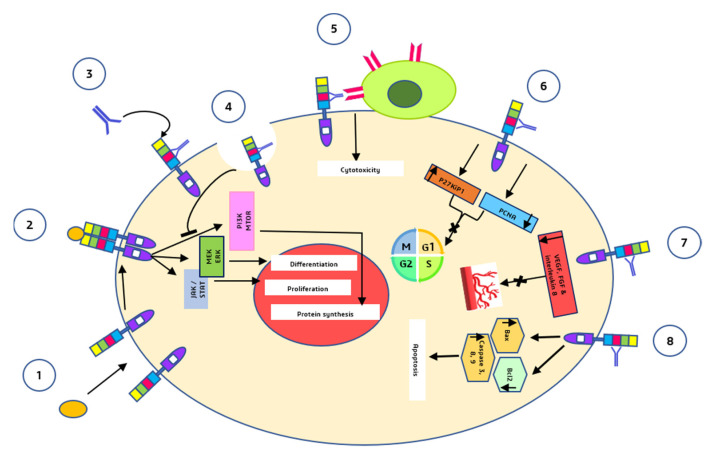
Major anti-tumor mechanisms by which cetuximab acts (1) and (2) are the mechanisms that normally take place in the absence of cetuximab i.e., binding of the ligands to the EGFR monomers, induction of receptor dimerization, and downstream signalling pathways. (3) Cetuximab binds to domain III of EGFR. (4) Receptor internalization mediated by cetuximab and inhibition of downstream signalling pathways. (5) Fc segment of cetuximab binds to natural killer cells and induces ADCC. (6) G1 cell cycle arrest. (7) inhibition of angiogenesis. (8) Induction of apoptosis.

Cetuximab (ERBITUX) is an FDA-approved chimeric (human–murine) IgG1 monoclonal antibody that competitively binds the extracellular domain of EGFR with a high affinity [10,29]. Since 2004, cetuximab has been approved as a single drug or in combination with irinotecan for the treatment of metastatic colorectal cancer with EGFR overexpression in patients with chemotherapy-resistant malignancies [34]. Cetuximab’s capacity to obstruct the EGFR pathway has been demonstrated in preclinical and clinical research. It has been shown in preclinical studies that cetuximab alone possesses cytostatic action; however, the use of cetuximab in combination with other chemotherapeutic drugs (such as platinum-derived compounds and irinotecan) enhances the antitumoral activity of the individual regimens. One explanation for this synergy is that limiting EGFR signaling is insufficient for cytotoxicity in the majority of cell lines, yet inhibiting EGFR makes cells more sensitive to chemotherapy [32]. Cetuximab is metabolized and eliminated by the reticuloendothelial system. Its clearance is unaffected by kidney or liver function; therefore, its pharmacokinetics are the same in people with normal or impaired renal function [35]. Cetuximab’s anticancer actions are mediated by a variety of pathways (Figure 3).

- Cetuximab binds to the second (L2) EGFR domain and consequently blocks downstream signaling by triggering receptor internalization and blocks the interaction between ligand and receptor [36].
- Through antibody-dependent cell-mediated cytotoxicity (ADCC), cetuximab directs cytotoxic immune effector cells toward EGFR-expressing tumor cells, potentially contributing to its antitumoral impact [37].
- Cetuximab causes a G1 cell cycle arrest by increasing the cell cycle inhibitor p27^kip1^ and suppressing proliferating cell nuclear antigen (PCNA) [38].
- Cetuximab inhibits angiogenesis by restricting the production of pro-angiogenic factors such as interleukin-8, vascular endothelial growth factor (VEGF), and basic fibroblast growth factor (FGF) [39].
- Induction of apoptosis by cetuximab is mediated through two processes: (a) Increased expression of pro-apoptotic proteins such as BAX, caspase-3, caspase-8, and caspase-9 and (b) inactivation of Bcl-2, which is an anti-apoptotic protein [39].

## 5. EGFR Targeted Therapy Using Cetuximab

Cetuximab-modified nanoparticles have the potential to act as targeting moieties for recognizing cells with EGFR-overexpression, among other diagnostic and therapeutic applications [40]. Table 2 compiles the collection of cetuximab conjugated nanocarriers.

### 5.1. Gold Nanoparticles

Studies investigating metallic nanoparticles (MNPs), such as gold nanoparticles, in EGFR-targeted therapies have shown that they can produce synergistic treatments, enhance therapeutic benefits, and delay the emergence of drug resistance. The pharmacokinetics and delivery of EGFR drugs can therefore be enhanced by AuNPs [41]. Because of their large surface area for drug attachment, AuNPs can be effective carriers for EGFR antibody and tyrosine kinase inhibitors in molecular-targeted therapy [42].

It has been demonstrated that gold nanoparticles conjugated to cetuximab can regulate tight junctions and mediate the death of cells in invasive colorectal carcinoma. In [44] examined the cytotoxicity and found that AuNPs 60 nm in diameter had the most impact on the viability of HT-229 cells. When cetuximab was coupled with AuNPs, three cell surface biomarkers, namely melanoma cell adhesion molecule, human epidermal growth factor receptor-3 (HER-3), and epithelial cell adhesion molecule (EpCAM), were detected to be expressed with increased heterogenicity [44].

### 5.2. Iron Oxide Nanoparticles

Nanotherapeutic systems based on iron oxide nanoparticles (IONPs) have a long history of clinical use for cancer treatment. IONPs are frequently employed in photodynamic therapy, laser-induced thermal ablation, radiotherapy, and magnetic resonance imaging (MRI) due to their unique magnetic and optical properties [27,42]. Numerous EGFR-targeted composite nanoplatforms containing IONPs as the primary component have been investigated thus far for MRI and targeted therapy. Cetuximab-conjugated magneto-fluorescent nanoparticles induced significant MRI signal changes in a human colon cancer xenograft mice model, and the introduction of an external magnetic field enhanced the local concentration of MFSN-Ctx in a tumor [45].

### 5.3. Polymeric Nanoparticles

With regards to their nanoscale size, capability for selective targeting, and controlled drug release, polymeric nanoparticles (NPs) appear to be highly promising drug carriers. Anticancer proteins are covalently conjugated to polymers, which decreases their immunogenicity while increasing their stability and blood circulation time [57]. It is then possible to deliver an anticancer treatment to a tumor site for an extended period using targeted delivery systems based on polymeric nanoparticles [58].

According to findings by Abdellatif et al., at pH 7.4, calcium alginate beads loaded with cetuximab-octreotide can specifically target an area in the GI tract, allowing cetuximab-octreotide to penetrate cells that express somatostatin receptors and subsequently dissociate into octreotide and cetuximab [59]. Caco2 cells overexpressing the epidermal growth factor receptor (EGFR) showed significantly greater anti-cancer activity than that of cells from the HCT-116 cell line. This difference can be attributed to the high expression of functional EGFR in Caco2 cells and the increased uptake of nanoparticles in cells by cetuximab-conjugated curcumin [47]. In a study by Maya et al., cetuximab-conjugated polyglutamic acid nanoparticles with loaded docetaxel (CET-DTXL-γ-PGA NPs) exhibited a twofold increase in nanoparticle absorption in HT-29 cells (EGFR+ve) compared to that of IEC-6 cells (EGFR-ve). Compared to non-conjugated nanoparticles, CET-DTXL-γ-PGA NPs demonstrated greater cytotoxicity, antiproliferative activity, and accelerated cell death due to cell cycle arrest at G2/M phase. Temozolomide-loaded poly (lactic-co-glycolic acid) nanoparticles (PLGA NPs) coupled with cetuximab showed better cellular uptake, greater cytotoxicity, increased apoptotic impact, and elevation of -H2A. The enhanced activity was attributed to EGFR-mediated endocytosis of cetuximab-conjugated PLGA NPs by EGFR-overexpressed malignancies [49].

### 5.4. Protein Nanoparticles

Protein nanoparticles are being investigated as viable delivery vehicles for a variety of therapeutic molecules such as growth factors, nucleic acids, therapeutic drugs, and vaccines. The primary goal of employing protein nanoparticles in cancer treatment is to increase therapeutic efficacy while minimizing negative effects. Surface modification of protein nanoparticles is used to provide site-specific medication delivery [60].

Cetuximab-valine-citrulline (vc)-doxorubicin immunoconjugates-loaded bovine serum albumin (BSA) nanoparticles prolonged doxorubicin absorption into cells and rendered the drug less susceptible to efflux effects. It also reduced systemic toxicity by improving drug accumulation in tumors rather than normal organs [50].

Cetuximab-conjugated bovine serum albumin nanoparticles loaded with doxorubicin demonstrated a significant in vitro cytotoxicity effect against EGFR-overexpressing colon cancer cells (RKO cells) compared to EGFR-under-expressing colon cancer cells (LS174 T cells), as well as decreased doxorubicin efflux in cetuximab-conjugated BSA nanoparticles. This study discovered that the conjugation of cetuximab to doxorubicin-loaded BSA nanoparticles improved selectivity while reducing toxicity [61].

### 5.5. Liposomes

For targeted drug delivery, liposomes are the most widely used and well-researched nanocarriers. Liposomes have several advantages as a drug delivery system including self-assembly capability, biocompatibility, the potential to transport large drug payloads, and a variety of physicochemical and biophysical features that may be manipulated to influence their biological characteristics. Liposomes that target specific ligands (e.g., receptors or adhesion molecules) at the site of the disease have a great deal of potential for site-specific delivery of drugs to specific cell types or organs in vivo [62].

Cetuximab-oxaliplatin liposomes were employed for targeted treatment of colorectal cancer in a study by Zalba et al. [52]. In cytotoxicity assays, targeted liposomes efficiently delivered intracellular oxaliplatin (L-OH), and L-OH resistance was reversed in two cell lines with decreased L-OH sensitivity when targeted liposomes were utilized. The anticancer activity of Fab’ ligand-targeted liposomes was shown to be more effective than that of cetuximab-targeted liposomes [52].

Previous authors synthesized cetuximab-decorated porphyrin-containing liposomal nanohybrid cerasomes (EGFR-CPIG), discovering that the EGFR-CPIG system accumulates preferentially at tumor locations and has a significant capacity for tumor targeting due to the combination of the EPR effect and the active tumor-targeting capability of EGFR [53].

### 5.6. Micelles

Polymeric micelles have received considerable interest because of their potential to protect bioactive payloads in their compartmentalized structures and release them when specific stimuli are detected, as well as their ability to accumulate selectively and significantly in tumor tissues. To target tumors, polymeric micelles should have a size between 10 and 100 nm, which reduces their accumulation in the reticuloendothelial system (RES) organs and allows them to bypass physiological barriers en route to their destination, including impactful extravasation and deep penetration in solid tumors after systemic injection. Additionally, increased cellular selectivity or micelle translocation across the impermeable endothelia of certain types of tumors can be obtained by coating them with ligand molecules that recognize specific epitopes on target cells [62].

Shin et al. used IR-780 iodide, a near-infrared dye, in micelles conjugated with the anti-EGFR antibody cetuximab for improved photothermal therapy. HCT-116 cells expressing a high level of EGFR were shown to have greater cellular uptake than SW-620 cells. The near-infrared fluorescence signals received by HCT-116 tumors were stronger than those received by SW-620 cancers. Higher photothermal therapy efficacy in HCT-116 tumors was attributed to increased IR 780 iodide delivery, which was facilitated by EGFR-targeted therapy [54].

### 5.7. Carbon Nanotubes

Carbon nanotubes (CNTs) primarily take the form of tubes or cylinders. They can vary in length, diameter, layer count, and chirality. They possess appealing features due to their unusual blend of strength, stiffness, and elasticity [63]. Due to their outstanding physical and chemical qualities, such as an enormous surface-area-to-volume ratio, hollow structure, small diameter, electrical capabilities, and exceptional optical and high tensile strength, CNTs are used in a wide range of applications. Using a CNT carrier, drug molecules can be delivered to cancer cells or diseased areas of the body with pinpoint accuracy and few side effects. CNTs can be fabricated with a tumor-targeting ligand to easily discriminate tumor cells from healthy cells for optimal targeting [64].

Single-walled carbon nanotubes (SWNTs) were coupled to the topoisomerase I inhibitor (SN38) and anti-EGFR antibody (cetuximab) for the delivery of SN38 to colon cancer cells by Lee et al. SWNT25/Py38 uptake was lower in the absence of the antibody than in the presence of the antibody, and the greatest uptake of SWNT25/Py38 was found in HCT-116 rather than HT29 or SW-620. Clathrin-dependent receptor-mediated endocytosis was discovered to be the route by which SWNT25/Py38 was taken up [55].

### 5.8. Quantum Dots

Quantum dots (QDs), due to their unique optical and electronic characteristics, are being intensively investigated as a novel probe in biomedical imaging in vitro as well in vivo. In order to overcome the challenges of QDs in biomedical imagery, extensive research has been conducted on their physicochemical properties, including their size, shape, composition, and surface features. QD-based probes conjugated with bimolecular compounds such as antibodies or peptides can be used to target small molecules of cancer with high specificity [65].

Hashemkhani et al. [56] used cetuximab-conjugated Ag_2_S-2MPA (AS-2MPA) quantum dots that emit NIR emission to act as theranostic agents in targeted ALA-PDT treatment and chemotherapy combination therapy of EGFR-positive colorectal cancer. In vitro studies in 2D and 3D cell cultures have shown expression-level-dependent EGFR targeting and release of ALA from AS-2MPA-ALA-Cet QDs, i.e., SW480 > HCT-116 > HT29, and there is also a strong intracellular NIR signal that contrasts well with the background [56].

## 6. Conclusions

The EGFR receptor has roles in the development of different epithelial cancers. This receptor is involved in proliferation, survival, and transformation. As a result, considerable effort has gone into developing and approving potent and selective anti-EGFR inhibitors, particularly monoclonal antibodies, which carry many benefits, including selectivity. Antibody-conjugated nanocarriers are a rapidly growing field of research with enormous clinical promise. Despite the significant effort put into their development and initial success, mAb-conjugated nanocarriers confront numerous challenges and limits. The targeting moiety is necessary for the system’s functionality. Amino acid changes in EGFR epitopes may alter the affinity of the mAb for the receptor, encouraging the development of new anti-EGFR antibodies. Approaches that use EGFR-targeted nanoparticles (active targeting) may have additional advantages over those that do not (EPR-based passive targeting). Because EGFR overexpression is found in 25 to 82% of colorectal cancer cells, cetuximab-modified nanoparticle carriers could be used as targeting moieties for detecting colorectal cancer cells with EGFR overexpression, as well as for other diagnostic and therapeutic purposes. The extent of receptor expression and the number of ligands required for successful targeting should be established in current and future research in this area. While mouse models are important, variations in murine and human biology, particularly nanocarrier clearing routes, are expected to pose challenges in the transition to promising human therapies. Using antibody-conjugated nanocarriers to achieve therapeutic success is thus an appealing endeavor.

## Figures and Tables

**Table 1:** List of EGFR monoclonalantibodies used in the treatment of colorectal cancer.

Name of the Antibody	Antibody Type	Disease	Molecular Weight	Mechanism of Action	Market Status	Side Effects
Cetuximab (Erbitux)	Chimeric antibodyIgG1	Head and neck cancer Metastatic colorectal cancer	145.7816 kDa	(1) Binds to domain III of EGFR receptor and prevents the conformational change required for EGFR activation (2) Induce apoptosis and decrease matrix metalloproteinase and VGEF	Marketed	rash, itching, dry or cracked skin, nail changes, headache, diarrhea, nausea, vomiting, upset stomach, weight loss, weakness, and respiratory, skin, and mouth infections.
Panitumumab (Vectibix)	Humanan tibody IgG2	Metastatic colorectal cancer	147 kDa	(1) Binds to domain III of EGFR receptor and prevents the conformational change required for EGFR activation	Marketed	Skin reactions, Fatigue, General deterioration, Abdominal pain, Nausea, Diarrhea, Vomiting, Swelling in hands or feet, Cough, Dry skin, Inflammation of the bed of the fingernails, Eye irritation

**Table 2:** List of Cetuximab conjugated carriers and their significance.

Carriers	Formulation	Method of Antibody conjugation/coating	Drug	Target cells/Animal models	Disease/Application	Significance
Gold nanoparticles	Tetrachloroauric acid, sodium citrate, and cetuximab	Physical adsorption	-	Caco-2, HT-29 and HCT-116	Colorectal cancer	Tight junction modulation by Gold nanoparticles aid in drug delivery. Improved cell death mediated by cetuximab [43].
Gold nanoparticles	Tetrachloroauric acid, sodium citrate, s 5,50 -dithiobis-(2-nitrobenzoic acid), 7-mercapto4-methyl coumarin, 2,3,5,6-tetrafluoro-4-mercaptobenzoic acid, and cetuximab	Physical adsorption	-	HT-29	Colorectal cancer	Expression of cell surface biomarkers such as MCAM, HER3, and EpCAM with more heterogenicity [44].
Magneto fluorescent silica nanoparticles	Polyvinylpyrrolidone, ferrite, 2- [methoxy- (polyethyleneoxy)propyl] trimethoxysilane, (3-trimethoxysilil) propyl diethylene triamine, and cetuximab antibody fragment	Physical adsorption	-	HCT 116, H520 cells, HT 29, SW620, and BALB/c nude male mice	Colorectal cancer	Considerable MRI signal changes Local concentration of MFSN-Ctx amplified by an external magnetic field [45].
Ca-alginate-beads	sodium alginate, calcium chloride, Octreotide, and Cetuximab	solvent evaporation method	Octreotide	MCF-7, HepG-2, and HCT-116	Breast cancer, Hepatocellular carcinoma, and colorectal cancer	Target a specific area in Gastro-Intestinal Tract Specifically penetrates somatostatin expressing cells and releases octreotide [46].
Chitosan-Pectin nanoparticles	Chitosan, pectin, curcumin and cetuximab	EDC–NHS chemistry	Curcumin	Caco-2, and HCT-116.	Colorectal cancer	EGFR overexpressing cancer cell line had stronger anti-cancer activity Cetuximab enhanced the nanoparticle uptake [47].
Γ Poly (glutamic Acid) nanoparticles	Γ Poly (glutamic Acid), chitosan, docetaxel, rhodamine-123, and cetuximab.	EDC–NHS chemistry	Docetaxel	HT-29, IEC-6, and Swiss Albino mice	Colorectal cancer	A two-fold increase in nanoparticle uptake in EGFR+ve cells increased cytotoxicity antiproliferative activity due to cell cycle arrest at G2/M phase [48].
PLGA nanoparticles	Poly (lactic-co-glycolic acid), Temozolomide, and cetuximab.	EDC–NHS chemistry	Temozolomide	U-87MG, SK-Mel 28, and SW480	Brain cancer, Skin cancer, and Colorectal cancer	Improved cellular uptake Increased cytotoxicity Increased apoptotic impact Upregulation of γ-H2A [49].
BSA nanoparticles	MC-Val-Cit-PAB-PNP, Doxorubicin, and cetuximab	Direct coupling	Doxorubicin	RKO, d LS174, and BALB/c nude mice	Colorectal cancer	Increased the duration of doxorubicin uptake into cells Improved accumulation of the drug in tumors Lowered systemic toxicity [50].
BSA nanoparticles	BSA, doxorubicin, and cetuximab	Direct coupling	Doxorubicin	RKO and LS174 t	Colorectal cancer	Significant cytotoxic activity of doxorubicin in EGFR overexpressing cells with increased selectivity and low toxicity [51].
Liposomes	Oxaliplatin, and Fab of cetuximab	Maleimide chemistry	Oxaliplatin	HCT-116, HT-29, SW-480, SW-620, and nude mice	Colorectal cancer	Effective delivery of intracellular L-OH Resistance to L-OH was reversed when targeted liposomes are used Fab’ ligand targeted liposomes were more efficacious in antitumor activity than cetuximab targeted liposomes [52].
Cerosomes	1,2-Distearoyl-sn-glycero-3- phosphoethanolamine-n-[poly(ethylene glycol)]-hydroxy succi- nimide, cetuximab, and porphyrin	Direct Coupling	Porphyrin	CT26-fLuc, and Balb/c mice	Colorectal cancer	Preferential accumulation of EGFR-CPIG at tumor locations The combination of EPR effect and the active tumor targeting capability of EGFR increase the tumour targeting capacity [53].
Micelles	ε-caprolactone, Methoxy poly ethylene glycol, Poly ethylene glycol monoethyl ether maleimide, (DTPA dianhydride, and cetuximab	Chemical modification (Thiolation)	IR-780 iodide (Near infrared dye)	HCT-116, SW-620, and nude mice	Colorectal cancer	Highest contrast of NIRF signals was obtained between HCT-116 and SW-620 tumors. Increased delivery of IR-780 and enhanced photo thermal therapy was observed in HCT-116 tumors (EGFR +ve ) [54].
Carbon nanotubes	Carbon nanotubes, SWNT-COOH, NH2-PEGNH2, and cetuximab	EDC–NHS chemistry	SN38 (Topoisomerase I inhibitor), and Pyrene 38	HCT116, HT29, and SW-620	Colorectal cancer	The targeting ability and delivery of SN38 by Cetuximab conjugated SWNT25/py38 was enhanced in EGFR +ve colorectal cancer cell line. Clatherin-dependent endocytosis was responsible for uptake of SWNT25/py38 [55].
Quantum Dots	Silver nitrate,5-aminolevulinic acid hydrochloride, 2-Mercaptopropionic acid, Sodium sulfide, And cetuximab	EDC–NHS chemistry	ALA	HCT116, HT29, and SW-480	Colorectal cancer	The targeting ability of quantum dots was observed to increase with increased EGFR expression and there is a strong intracellular NIR signals [56].

## Data Availability

Not applicable.
